# Treatment of patients with atypical meningiomas Simpson grade 4 and 5 with a carbon ion boost in combination with postoperative photon radiotherapy: The MARCIE Trial

**DOI:** 10.1186/1471-2407-10-615

**Published:** 2010-11-09

**Authors:** Stephanie E Combs, Lutz Edler, Iris Burkholder, Stefan Rieken, Daniel Habermehl, Oliver Jäkel, Thomas Haberer, Andreas Unterberg, Wolfgang Wick, Jürgen Debus, Renate Haselmann

**Affiliations:** 1 Department of Radiation Oncology, University Hospital of Heidelberg, Im Neuenheimer Feld 400, 69120 Heidelberg, Germany; 2 Institute of Medical Biometry and Informatics, University of Heidelberg, Im Neuenheimer Feld 305, 69120 Heidelberg, Germany; 3 Heidelberger Ionenstrahl Therapiezentrum (HIT), Im Neuenheimer Feld 450, 69120 Heidelberg, Germany; 4 Department of Neurosurgery, University Hospital of Heidelberg, Im Neuenheimer Feld 400, 69120 Heidelberg, Germany; 5 Department of Neurooncology, University Hospital of Heidelberg, Im Neuenheimer Feld 400, 69120 Heidelberg, Germany; 6 StaBiL, Statistische und Biometrische Lösungen, Pistorstr. 7, 66482 Zweibruecken, Germany

## Abstract

**Background:**

Treatment standard for patients with atypical or anaplastic meningioma is neurosurgical resection. With this approach, local control ranges between 50% and 70%, depending on resection status. A series or smaller studies has shown that postoperative radiotherapy in this patient population can increase progression-free survival, which translates into increased overall survival. However, meningiomas are known to be radioresistant tumors, and radiation doses of 60 Gy or higher have been shown to be necessary for tumor control.

Carbon ions offer physical and biological characteristics. Due to their inverted dose profile and the high local dose deposition within the Bragg peak precise dose application and sparing of normal tissue is possible. Moreover, in comparison to photons, carbon ions offer an increased relative biological effectiveness (RBE), which can be calculated between 2 and 5 depending on the cell line as well as the endpoint analyzed.

First data obtained within the Phase I/II trial performed at GSI in Darmstadt on carbon ion radiotherapy for patients with high-risk meningiomas has shown safety, and treatment results are promising.

**Methods/design:**

The Phase II-MARCIE-Study will evaluate a carbon ion boost applied to the macroscopic tumor in conjunction with photon radiotherapy in patients with atypical menigiomas after incomplete resection or biopsy.

Primary endpoint is progression-free survival, secondary endpoints are overall survival, safety and toxicity.

**Discussion:**

Based on published data on the treatment of atypical meningiomas with carbon ions at GSI, the present study will evaluate this treatment concept in a larger patient population and will compare outcome to current standard photon treatment.

**Trial registration:**

NCT01166321

## Background

Meningiomas are the second most common primary brain tumor and represent approximately 15-26% of all intracranial neoplasms [1]. Approximately 5-10% of meningiomas are of non-benign histology (atypical or anaplastic) and are associated with less favourable outcome; they are characterized by locally aggressive growth and early recurrence or tumor progression after surgery [2]. Therefore, neurosurgical resection alone does not confer to high long-term local control and overall survival rates [3]. Moreover, extent of tumor resection has shown to be a significant prognostic factor. The resection level is commonly assessed after neurosurgical resection by the surgeon after verification with a postoperative MRI and is classified according to Simpson guidelines (see table 1).

**Table 1:** Simpson grading for Meningioma

Radical	
**Stage 1**	complete excision, including dura and bone
**Stage 2**	complete excision + supposed reliable coagulation of dural attachment.
**Non-radical**	
**Stage 3**	complete excision but insufficient dural coagulation or bone excision (non visible on MR, according to surgeon's opinion)
**Stage 4**	incomplete excision, macroscopic rest visible (on MRI)
**Stage 5**	biopsy only (visible on MRI)

Patients after non-radical resection (Simpson Grade 4 and 5) show significantly worse outcome than patients after radical neurosurgical resection [4]: In patients with non-bening meningiomas treated with surgery alone, local recurrence rates are 50% for subtotally excised, and 90% for completely resected patients at 3 years as reported in a large multi-center overview. Local recurrence has a major negative impact on survival.

Although meningiomas are classified as relatively radioresistant tumors, radiation therapy is the most effective adjuvant treatment available [5-8]. For non-benign meningiomas, a number of small and non- controlled series have reported superior outcome after postoperative radiotherapy as compared to surgery alone [6,9-13].

Novel radiotherapeutic modalities such as protons or carbon ion radiotherapy offer a promising treatment alternative. Radiation therapy using charged particles is characterized by distinct physical and biological characteristics. Charged particles provide the physical advantage of an inverted dose profile which enables steep dose gradients. Therefore, reduction of dose to normal tissue, and especially to organs at risk, can be achieved. Heavy charged particles, such as carbon ions, additionally offer an increased RBE. Thus, with particle therapy, dose escalation required for long-term control of atypical or malignant meningiomas is feasible while adhering to normal tissue tolerance constraints. New beam qualilties, such as carbon ion radiation therapy, offer a promising treatment alternative in malignant meningiomas. Carbon ion radiotherapy offers the physical characteristics as do protons, however, are characterized by a comparably higher RBE. For aggressive and radioresistant tumors auch as glioblastoma cell lines, RBE values between 2 and 5 have been reported depending on cell line and endpoint [14,15]. Clinically, it has been shown that carbon ion radiation therapy leads to significantly increased control rates in treatment-resistant tumors [16-18].

Carbon ion radiotherapy was available by the Department of Radiation Oncology at GSI in Darmstadt since 1997. Superior treatment results for a number of tumor entities, such as chordomas and chondrosarcomas of the skull base, as well as ACC have been shown, and carbon ion radiotherapy is currently performed in the clinical routine for these patients [17,18]. Safety of carbon ion radiotherapy with respect to critical organs at risk, such as the brain, brainstem or spinal chord, have been shown in these studies. At the HIT center, treatment of over 1300 patients per year with Proton and Carbon ion RT is possible. Worldwide, particle therapy is available in the clinical routine in a few of centers until now.

Over recent years, a number of centers have implemented proton radiotherapy for patients with meningiomas, mainly with benign histology. Results are summarized in table 2.

**Table 2:** Results of Proton and Carbon Ion Therapy for Patients with Meningiomas

Author	Institute	Year	No. of Pts.	Radiation Modality	Overall Survival	Local Control
Austin-Seymour et al.	MGH, Boston MA, USA	1990	13	Protons, 59,4 Gy E	100%	100%
Gudjonsson et al.	Uppsala, Sweden	1999	19	Protons, 24 Gy E, 4 fractions	-	100% at 3 years
Wenkel et al.	MGH, Boston MA, USA	2000	46	Protons and Photons, 59.0 Gy E	95% and 77% at 5 and 10 years	100% and 88% at 5 and 10 years
Hug et al.	MGH, Boston MA, USA	2000	16	Protons and Photons, 62 Gy E for atypical meningiomas, 58 Gy E for malignant meningiomas	-	19% at 8 years for atypical meningiomas, 17% at 8 years for malignant meningiomas
Vernimmen et al.	Tygerberg, South Africa	2001	27	Protons, 54 Gy E - 61.6 Gy E, 16 - 27 fractions	-	88%
Noël et al.	CPO, Orsay, France	2002	17	Protons and Photons, 61 Gy E	88.9% at 4 years	87.5% at 4 years
Weber et al.	PSI, Switzerland	2004	16	Protons, 52.2-64 Gy E	92.7% at 3 years	91.7% at 3 years
Noël et al.	CPO, Orsay, France	2005	51	Protons and Photons, 60.6 Gy E	100% at 4 years	98% at 4 years
Combs et al.	Heidelberg, Germany	2010	10	Photons, Carbon Ion Boost	75% and 63% at 5 and 7 years	86% and 72% at 5 and 7 years

Outcome after proton as well as advanced photon radiotherapy are comparable for the treatment of benign meningiomas, with high local control rates and low rates of treatment-related morbidity. However, for benign meningiomas, total doses of 50 to 60 Gy are required for long-term tumor control. For anaplastic and atypical meningiomas, on the other hand, doses of 60 Gy are known to be not sufficient for long-term tumor control, and possibilities of dose-escalation play an important role for the improvement of outcome. Smaller studies could show that total doses exceeding 60Gy are required for long-term tumor control [13,19]. Therefore, until now, distinctly worse clinical results are still observed observed in WHO Grade II and III meningiomas. Atyical and anaplastic meningiomas remain a difficult to treat patient population with high rates of local tumor recurrences, even after aggressive surgery and adjuvant radiation therapy.

Preliminary results obtained after carbon ion radiotherapy within a Phase I/II trial performed at GSI could show safety and feasibility in patient with high-risk meningiomas treated with a carbon ion boost to the macroscopic tumor in combination with precision photon radiotherapy delivered as IMRT or FSRT [20]. These patients had been treated in our institution within a dose of 50.4 Gy with photons delivered by FSRT or IMRT, and a carbon ion boost to the macroscopic tumor with a total dose of 18Gy E in single doses of 3 Gy E [20]. No severe acute or long-term toxicity could be observed, and treatment outcome with with overall survival rates of 75% and 63% at 5 and 7 years is promising, although all patients had macroscopic tumors at the timepoint of radiotherapy. Therefore, the concept of a carbon ion boost to patients with atypical meningiomas with a macroscopic tumor lesion after neurosurgical resection is a promising treatment alternative.

In the present MARCIE trial, impact of a carbon ion boost using intensity modulated rasterscanning delivered in combination with precision photon radiotherapy will be evaluated in a Phase II design in patients with atypical meningiomas.

## Methods and Design

The purpose of the trial is to evaluate a carbon ion boost to the macroscopic tumor in combination with photon radiotherapy in patients atypical meningiomas.

The aim of the study is to compare progression-free survival as a primary endpoint, and overall survival free survival, toxicity and safety as secondary endpoints.

The primary objective is progression-free survival rate during the follow-up phase of at least 3 years. The secondary objectives of the study are overall survival, toxicity and safety.

The trial will be performed as a single-center one-armed Phase II study.

Patients fulfilling the inclusion criteria will be included.

### Treatment Schedule

Carbon Ion Radiation Therapy as a Boost to the macroscopic tumor

Total Dose 18 Gy E, 6 fractions, 3 Gy E single dose

**Figure 1:**
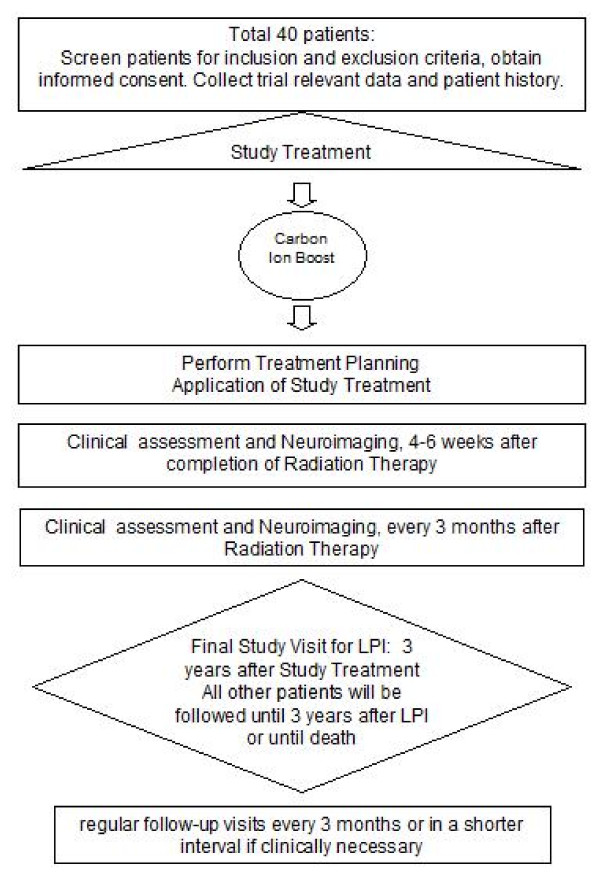
**Flow chart of the MARCIE-Study**.

The trial workflow is shown in Figure 1.

### Study objectives and endpoints

The primary endpoint is progression-free survival at 3 years, therefore patients are followed within the trial protocol for a minimum 3 years after completion of study treatment. For the LPI (last patient in), the final study visit will be 3 years after study treatment to asses the primary endpoint. All other patients will be followed on a regular basis as stated below until death or until 3 years after LPI.

After RT, patients are scheduled for follow-up visits every 3 months or as needed clinically including contrast-enhanced MRI as well as thorough clinical-neurological assessment.

The last patient included into the study will be followed 3 years after treatment. This is considered the final study visit. All other patients will be followed regularly as described in detail until death or until 3 years after LPI.

The overall duration of the trial is expected to be approximately 72 months. Recruitment of the patients is planned over a time period of 36 months, minimum duration of the follow-up phase will be 36 months.

### Patient selection

40 patients should be enrolled in this clinical study fulfilling the following inclusion criteria.

### Inclusion criteria

Patients meeting all of the following criteria will be considered for admission to the trial:

- histologically confirmed atypical meningioma

- macroscopic tumor after biopsy or subtotal resection - Simpson Grade 4 or 5

- prior photon radiotherapy to the CTV_Photon _of 48-52 Gy

- beginning of study treatment no later than 12 weeks after surgery

- age ≥ 18 years of age

- Karnofsky Performance Score ≥60

- For women with childbearing potential, adequate contraception.

- Ability of subject to understand character and individual consequences of the clinical trial

- Written informed consent (must be available before enrolment in the trial)

### Exclusion criteria

Patients presenting with any of the following criteria will not be included in the trial:

- refusal of the patients to take part in the study

- previous radiotherapy of the brain

- time interval of >12 weeks after primary diagnosis (neurosurgical intervention) and beginning of study treatment

- optic nerve sheath meningioma (ONSM)

- Patients who have not yet recovered from acute toxicities of prior therapies

- Known carcinoma <5 years ago (excluding Carcinoma in situ of the cervix, basal cell carcinoma, squamous cell carcinoma of the skin) requiring immediate treatment interfering with study therapy

- Pregnant or lactating women

- Participation in another clinical study or observation period of competing trials, respectively.

### Treatment Assignment

All Patients will be assigned the same treatment regimen within this study

### Treatment Planning

For radiotherapy, patients will be immobilized using an individually manufactured head mask. For treatment planning, contrast-enhanced CT as well as MR-imaging will be performed for optimal target definition.

Organs at risk such as the brain stem, optic nerves, chiasm and spinal chord will be contoured. Dose constraints of normal tissue will be respected according to Emami et al. [21].

The carbon ion boost will consist of the GTV including the area of contrast enhancement on T1-weighted MR-imaging; the CTV_Carbon _for the boost will be defined adding a safety margin of 5 mm.

Amino-Acid-PET or ^68^Ga-Dotatoc-PET may be used in addition to contrast-enhanced MRI for target volume definition but is not mandatory.

For photon treatment, the CTV_Photon _will be defined, which is applied as 3D-conformal treatment or as high-precision photon radiotherapy such as FSRT or IMRT. The CTV_Photon _will be contured including the GTV and sub clinical microscopic tumor (e.g. including the pre-operative tumor or post-operative tumor bed, peritumoral edema, hyperostotic changes if any, and dural enhancement or thickening as seen in the CT/MRI at diagnosis) plus a 1 cm safety margin. The margin may be reduced with respect to anatomical borders or organs at risk at the discretion of the investigator. Treatment Planning for Photon Radiotherapy will be performed using the Planning Systems available at the Department of Radiation Oncology in Heidelberg, Germany (including HELAX-Masterplan/Nucletron, Virtuos-Konrad/Siemens, or STP/Stryker-Leibinger).

Carbon ion RT planning is performed using the treatment planning software PT-Planning (Siemens, Erlangen, Germany) including biologic plan optimization. Biologically effective dose distributions will be calculated using the a/ß ratio for meningioma as well as for the endpoint late toxicity to the brain.

No interruptions >4 days between the end of photon radiotherapy and the carbon ion boost are allowed.

Patient positioning prior to radiotherapy will be evaluated by comparison of x-rays to the DRRs. Set up deviations >3 mm are corrected prior to radiotherapy.

### Dose Prescription Carbon Boost

The intensity-controlled rasterscan system will be used for beam application. Six fractions of a single dose of 3 Gy E up to a total dose of 18 Gy E will be prescribed to the maximum of the calculated dose distribution for the target volume. Treatment planning aims at the coverage of the target volume by the 90%-isodose line.

Dose specification is based on biologic equivalent dose because of the high relative biologic effectiveness (RBE) of carbon ions, which differs throughout the target volume due to its dependence on various factors. RBE will be calculated at each voxel throughout the target volumes and biological optimization will be performed. The dose prescription used is related to the isoeffective dose GyE using daily fractions of 2 Gy and a weekly fractionation of 5 × 2 Gy.

### Follow-up

After completion of study treatment no further adjuvant treatment is scheduled or recommended. Any systemic treatment or chemotherapy or any other treatment applied is not part of the clinical trial.

For tumor progression, treatment alternatives will be evaluated and discussed in the interdisciplinary setting considering options of neurosurgical resection, systemic treatment such as chemotherapy, a second course of radiation therapy, or other.

This study will use the International Common Terminology Criteria for Adverse Events (CTCAE) version 4.0 for toxicity and adverse event reporting. A copy or the CTCAE can be accessed from the CTEP home page (http://ctep.cancer.gov/protocolDevelopment/electronic_applications/ctc.htm).

Safety and toxicity of the study treatment will be evaluated by clinical neurological examination as well as neuro-imaging studies (MRI or CT).

### - Progression-free Survival -

Progression-free survival is the primary endpoint of the study. Progression-free survival will be counted from the first day of radiotherapy treatment until the date of the first event of either progression or death due to any cause. Patients alive without progressive disease at the time of data analysis will be censored at the time of the most recent follow-up visit.

#### Complete remission

Remission of all contrast-enhancing lesions on CT or MRI without worsening of neurologic status

#### Partial remission

at least 50% remission of the contrast-enhancing lesions on CT oder MRI without increase in steroid medication and without worsening of the neurologic status

#### Stable disease

Remission of the solid tumor/contrast-enhancing lesion on CT or MRI of less than 50% or progression of the solid tumor/contrast-enhancing lesion on CT or MRI of less than 25%, without increase in steroid medication of worsening of the neurologic status

#### Progression

Increase in solid tumor/contrast-enhancing lesion of 25% or more or development of a new lesion

#### Progression-free survival will be assessed following the target lesion(s) as defined hereunder

The initial assessment of the disease (including CT and MRI) must be performed between neurosurgical intervention and the beginning of study treatment. Follow-up assessments (including MRI or CT) will be performed as described until disease progression (even after the end of the study).

Special attention should be given so as to avoid tissue reaction to radiation treatment to be classified as tumor or disease progression. Such variations in post-radiotherapy imaging may continue for months, and may be accompanied by clinical signs and symptoms. In addition, surgical procedures may cause increased contrast uptake which should be differentiated from tumor progression. The clinical follow-up must dictate how the initial progression of the lesion should be labeled. If the course of events shows that true progression indeed occurred, the date of the first increase is to be considered as the date of progression. The principal investigator or the study coordinator may be contacted for further discussion on a case by case basis.

### Radiological progression

The lesion must be measured in the two largest perpendicular diameters; the area should be defined as the product of these two diameters.

- Increase of the lesion on MRI or CT scans of more than 25% as measured by two perpendicular diameters compared to the smallest measurements ever recorded for the same lesion by the same technique.

- The appearance or new lesions with or without contrast enhancement.

### - Overall Survival -

Overall survival is the primary endpoint of the study. All patients will be followed until death. The duration of survival is the time interval between initial diagnosis (date of the neuropathology report) and the dated of death due to any cause. Patients not reported dead or lost to follow-up will be censored at the date of the last follow-up examination.

### Statistical Considerations

The statistical methods applied for this study are subject to GCP guidelines (Guidelines of the International Conference on Harmonisation (ICH) e.g.

• ICH E3: Structure and Contents of Clinical Study Reports,

• ICH E6: Good Clinical Practice (GCP). Consolidated Guideline,

• ICH E9: Note for Guidance on Statistical Principles in Clinical Trials) and will be performed in accordance with CESAR SOP 8 (Statistical Analysis/Biometry) in their versions valid at the date of the original study protocol.

### Study Hypothesis

The study is designed to demonstrate that a carbon ion boost in combination with postoperative photon radiotherapy can improve the progression-free survival rate after 3 years (PFS-3yR) by 20%. The benchmark for largest PFS-3yR which, if true, implies that the efficacy of study treatment is too low is assumed to be 50% according to literature data (4) with a comparable patient population (patients with atypical meningiomas Simpson grade 4 and 5 and without previous radiotherapy).

### Sample Size Calculation

The sample size calculation is based on the analysis of the primary endpoint PFS-3yR, denoting the rate of progression-free survival after 3 years of follow-up. The trial is designed to detect an improvement by 20% in this primary endpoint. For sample size calculation, the following hypotheses will be made:

• p_0 _is the largest PFS-3yR which, if true, implies that the efficacy of the treatment is too low. In the present trial p_0 _has been taken as 50%.

• p_1 _is the lowest PFS-3yR which, if true, implies that the efficacy of the treatment is adequate. In the present trial p_1 _has been taken as 70%.

• α is the accepted probability of considering adequate efficacy of the treatment with a true PFS-3yR equal or lower to p_0_. In the present trial α has been taken as 5% (actual 0.049).

• β is the accepted probability of rejecting adequate efficacy of the treatment with a true PFS-3yR at least equal to p_1_. In the present trial α has been taken as 20% (actual 0.0193).

Using the one-sided binomial test with the given hypotheses, the study required 37 patients to decide whether the PFS-3yR is less than or equal to 0.5 or greater than or equal to 0.7. If the number of progressions or deaths is lower than 13, the hypothesis that PFS-3yR ≤ 0.5 is rejected with a target error rate of 0.05 and an actual error rate of 0.049. Sample size calculation was done using the software PASS 2008.

Allowing for approximately 10% non-evaluable patients primarily due to drop out, n = 40 patients will be recruited.

Assuming the distribution of PFS events is exponential, PFS-3yR = 0.5 resp. PFS-3yR = 0.7 is equivalent to a median PFS = 3 years resp. median PFS = 5.8 years.

### Analysis Variables

The primary outcome variable is progression-free survival rate at 3 years. Secondary objectives are the assessment of overall survival as well as 'toxicity' and 'safety'.

### Analysis Populations

#### Full Analysis Population

The full analysis set (FAS) according to intention to treat (ITT) consists of all patients included in the trial irrespective whether any protocol violation was present at the time of treatment start or during treatment under study conditions or whether the patient withdrew consent or was taken off-study any time after treatment start.

Not included are patients who withdraw informed consent before start of treatment or about whom it becomes known that major in/exclusion criteria were violated which would have excluded them from study treatment when known at start of treatment.

#### Per Protocol Population

The per protocol population comprises only patients meeting eligibility criteria and receiving treatment according to this protocol.

#### Safety Population

All patients of the FAS receiving the study treatment at least once are part of the safety population.

### Statistical Methods

#### Confirmatory Analysis

The primary aim and its corresponding endpoint are subject to a confirmatory statistical analysis. The primary aim is to evaluate efficacy of carbon ion boost in combination with postoperative photon radiotherapy in patients with atypical meningiomas Simpson grade 4 and 5 on progression-free survival rate after 3 years of follow-up (PFS-3yR). Formally,

H_0_: PFS-3yR ≤ 0.5 versus H_1_: PFS-3yR > 0.5.

PFS-3yR is statistically tested with a one-sided binominal test on the level α = 5%.

#### Descriptive Analysis

Secondary endpoints are all of explorative nature and reported using descriptive analysis methods.

• For failure time data (progression-free survival and overall survival) Kaplan-Meier curves will be displayed and median estimates as well as associated 95% confidence intervals will be reported.

• Safety/tolerability will be assessed by the type, incidence, severity (graded by the NCT CTCAE Version 4.0), and relatedness of AEs to treatment and by assessment of all parameters related to safety. Tolerability and dosing will be described by numbers of patients in whom treatment was given as planned, delayed or permanently stopped.

### Data Management

According to the §13 of the German GCP-Regulation all important trial documents will be archived for at least 10 years after the trial termination.

According to the §28c of the German X-Ray Regulation (RöV) and the §87 of the German Radiation Protection Regulation (StrlSchV) the informed consent forms including patients' consent for trial participation, application of irradiation and data transmission to the competent authority will be archived for at least 30 years after the trial termination.

The Study Center at the Department of Radiation Oncology Define will be responsible for archiving all relevant data.

### Ethical and Legal Aspects

#### Good Clinical Practice

The procedures set out in this trial protocol, pertaining to the conduct, evaluation, and documentation of this trial, are designed to ensure that all persons involved in the trial by Good Clinical Practice (GCP) and the ethical principles described in the applicable version of the Declaration of Helsinki (2008 Version of the Declaration of Helsinki, adopted at the 59th WMA General Assembly, Seoul, October 2008).

The trial will be carried out by adhereing to local legal and regulatory requirements.

The study plan has obtained approval by the Institutional Review Board (IRB)/Independent Ethics Committee (EC) of the Medical Faculty Heidelberg as well as the Bundesamt für Strahlenschutz (BfS).

## Discussion

Treatment optimization for patients with high-grade meningiomas is a main goal of the radiation oncologist. It is known, that, for long-term local tumor control, high doses of radiotherapy are required. Previous studies have shown beneficial results for particle therapy in patients with meningiomas, however, most studies have evaluated proton radiotherapy in low-grade meningioma patients. Prior studies from our institution have shown that carbon ion radiotherapy can improve local tumor control in several tumor types [17,18]. For high grade meningioma patients, a combined treatment of photon radiotherapy and a carbon ion boost to the macroscopic tumor have proven safety and promising efficacy [20]. Therefore, this concept will be evaluated in the first prospective trial on carbon ion radiotherapy for atypical meningiomas.

## Abbreviations

ACC: adenoid cystic carcinomas; CT: computer tomography; CTCAE: Common Toxicity Criteria for Adverse Events; CTV: clinical target volume; DKFZ: Deutsches Krebsforschungszentrum; DRR: digitally reconstructed radiograph; EC: Ethics Committee; FSRT: Fractionated Stereotactic Radiotherapy; GCP: Good Clinical Practice; GSI: Gesellschaft für Schwerionenforschung; GTV: gross tumor volume; Gy: Gray; Gy E: Cobalt Gray equivalent; HIT: Heidelberg Ion Therapy Center; IMRT: Intensity Modulated Radiotherapy; ITT: Intention To Treat; LET: linear energy transfer; LLUMC: Loma Linda University Medical Center; MGH: Massachusetts General Hospital; MR: Minor responses; MRI: magnet resonance imaging; OS: Overall survival; PFS: progression-free survival; PFS-3yR: progression-free survival rate after 3 years of follow-up; PR: Partial response; RBE: relative biological effectiveness; SD: Stable disease; RT: radiation therapy; WHO: World Health Organisation

## Competing interests

The authors declare that they have no competing interests.

## Authors' contributions

SEC and JD have developed the study concept. SEC, JD, IB and LE wrote the study protocol and obtained ethics approval. SEC, SR, DH, JD, WW, AU and RH will provide patient care. TH and OJ will perform treatment planning and beam application for carbon ion radiotherapy. SEC, WW, JD, and RH will implement the protocol and oversee collection of the data. All authors contributed to and approved the final manuscript.

## Pre-publication history

The pre-publication history for this paper can be accessed here:

http://www.biomedcentral.com/1471-2407/10/615/prepub
